# Regeneration Through *in vivo* Cell Fate Reprogramming for Neural Repair

**DOI:** 10.3389/fncel.2020.00107

**Published:** 2020-04-24

**Authors:** Wenjiao Tai, Xiao-Ming Xu, Chun-Li Zhang

**Affiliations:** ^1^Department of Molecular Biology, University of Texas Southwestern Medical Center, Dallas, TX, United States; ^2^Hamon Center for Regenerative Science and Medicine, University of Texas Southwestern Medical Center, Dallas, TX, United States; ^3^Spinal Cord and Brain Injury Research Group, Stark Neurosciences Research Institute, Indianapolis, IN, United States; ^4^Department of Neurological Surgery, Indiana University School of Medicine, Indianapolis, IN, United States

**Keywords:** *in vivo* reprogramming, adult neurogenesis, traumatic brain injury (TBI), spinal cord injury (SCI), retinopathy, Alzheimer’s diseases (AD), Parkinson’s disease (PD)

## Abstract

The adult mammalian central nervous system (CNS) has very limited regenerative capacity upon neural injuries or under degenerative conditions. In recent years, however, significant progress has been made on *in vivo* cell fate reprogramming for neural regeneration. Resident glial cells can be reprogrammed into neuronal progenitors and mature neurons in the CNS of adult mammals. In this review article, we briefly summarize the current knowledge on innate adult neurogenesis under pathological conditions and then focus on induced neurogenesis through cell fate reprogramming. We discuss how the reprogramming process can be regulated and raise critical issues requiring careful considerations to move the field forward. With emerging evidence, we envision that fate reprogramming-based regenerative medicine will have a great potential for treating neurological conditions such as brain injury, spinal cord injury (SCI), Alzheimer’s disease (AD), Parkinson’s disease (PD), and retinopathy.

## Introduction

A functional central nervous system (CNS) consists of both neurons and glial cells. While neurons are responsible for generating and communicating electrical and chemical signals essential for neural networks, their activities are supported and modulated by surrounding glial cells (Rasband, 2016). Both neurons and glia can be affected by pathological conditions such as neural injuries and degenerative diseases. Disruption of functional neural networks is frequently permanent and is the underlying mechanism for many pathological symptoms for which no effective therapeutics exists. An unmet challenge is how to promote neural regeneration for CNS repair after various pathological conditions.

New neurons can be generated in the adult mammalian brain through a process of neurogenesis, which is positively or negatively regulated by pathological conditions (Zhao et al., 2008). However, innate neurogenesis is temporally and spatially restricted and is generally not sufficient for functional neural repair.

*In vivo* neural reprogramming is emerging as a promising new strategy for regenerative medicine (Chen et al., 2015; Smith and Zhang, 2015; Li and Chen, 2016; Smith et al., 2017; Torper and Götz, 2017; Barker et al., 2018; Wang and Zhang, 2018). This strategy employs genetic and epigenetic methods to reprogram resident glial cells into neuronal progenitors and mature neurons in living animals. Unlike neurons that are frequently lost in response to pathological conditions, glial cells rather become activated and can form glial scars (Dixon, 2017; Gitler et al., 2017; Hayta and Elden, 2018). Though reactive glial cells may initially play beneficial roles, their persistent activation and scar formation are in general believed to hinder neural regeneration and may cause secondary damage to the surrounding tissues (Oyinbo, 2011; Freire, 2012; Anderson et al., 2016). *In vivo* fate reprogramming may turn reactive glial cells into useful neurons for damaged tissues.

**Figure 1:**
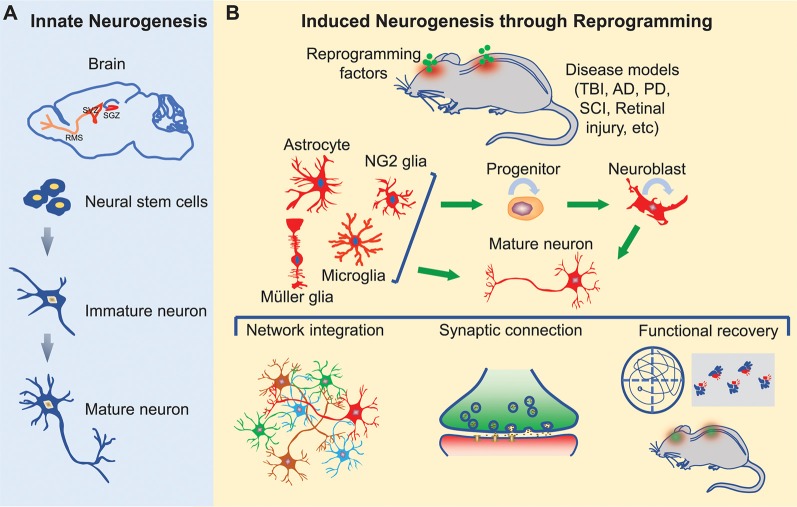
Innate and induced neurogenesis in the adult central nervous system. **(A)** Innate adult neurogenesis mainly occurs in the subgranular zone (SGZ) of the hippocampal dentate gyrus and the subventricular zone (SVZ) of the lateral ventricle. Neural stem cells in these regions generate neurons under physiological and certain pathological conditions. Neurons generated in the SGZ remain in the dentate gyrus, whereas neurons originated from SVZ migrate along the rostral migratory stream (RMS) into the olfactory bulb. **(B)** Induced adult neurogenesis occurs through cell fate reprogramming in multiple regions of the brain, spinal cord, and retina. Resident glial cells can be directly reprogrammed into mature neurons or progenitors. The induced progenitors can expand through proliferation and eventually give rise to mature neurons. These induced neurons may integrate into the neural networks and promote functional recovery following neural injury or degeneration.

Here, we will briefly review pathological regulations of innate neurogenesis and then focus on cell fate reprogramming for induced neurogenesis in the adult mammalian CNS (Figure 1).

## Pathological Modulation of the Innate Neurogenesis

Decades of research showed that neurogenesis persists in restricted adult brain regions of many mammalian species: the subventricular zone (SVZ) of the lateral ventricle and the subgranular zone (SGZ) of the hippocampal dentate gyrus (DG; Zhao et al., 2008; Ziemka-Nałecz and Zalewska, 2012; Obernier et al., 2018). The adult neurogenesis process could be modulated by neural injuries and pathological conditions.

Traumatic brain injury (TBI) triggers rapid cell death predominantly in the cerebral cortex followed by secondary tissue loss in the hippocampus. TBI-induced changes in adult neurogenesis are well observed in rodents (Urrea et al., 2007; Wang X. et al., 2016). Such changes are further shown to be dependent on TBI severity (Wang X. et al., 2016). Both mild and moderate TBI are not sufficient to induce new neurons through neurogenesis, even though moderate TBI can promote the proliferation of innate neural stem cells. Only severe TBI can lead to enhanced neurogenesis indicated by both neural stem cell proliferation and the formation of new neurons. Very interestingly, studies of 11 human brain specimens showed that TBI induced proliferation of cells expressing markers for neural stem cells in the perilesional cortex of human brains, although whether new neurons are generated remains to be determined (Zheng et al., 2013).

Disruption of adult brain neurogenesis may represent a critical feature in many neurological diseases. Epilepsy, for example, modulates many cellular steps of adult neurogenesis including stem cell proliferation and migration and integration of newborn neurons (Jessberger and Parent, 2015). Adult neurogenesis was observed to be enhanced in the hippocampus of human patients with Alzheimer’s disease (AD) and a mouse AD model (Jin et al., 2004a,b), whereas other mouse AD models rather showed decreased hippocampal neurogenesis (Donovan et al., 2006; Zhang et al., 2007). Adult hippocampal neurogenesis is similarly dysregulated in human Parkinson’s disease (PD) and mouse models of PD (Desplats et al., 2012; Winner et al., 2012; He and Nakayama, 2015).

Unlike the brain in which the phenomenon of adult neurogenesis is well-established, the spinal cord lacks any intrinsic ability to generate new neurons in adulthood (Horner et al., 2000). Spinal cord injury (SCI) can lead to the proliferation of multiple cell types including astrocytes and NG2 glia (also known as oligodendrocyte progenitor cells). However, none of these cells has been convincingly shown to generate mature neurons *in vivo* (Yamamoto et al., 2001; Horky et al., 2006). SCI also induces the proliferation of ependymal cells lining the central canal. When isolated and cultured *in vitro*, these cells exhibit stem cell-like properties that can form neurospheres and give rise to neurons, astrocytes, and oligodendrocytes (Meletis et al., 2008). Nevertheless, only astrocytes and oligodendrocytes are produced from injury-activated ependymal cells under *in vivo* conditions (Barnabé-Heider et al., 2010). It should be noted that, however, such a stem cell-like role of ependymal cells was recently questioned. The data from using more stringent genetic lineage tracing mouse lines rather showed that ependymal cells in either the adult brain or spinal cord lack any properties of stem cells and rarely contribute new cells to the injury site (Ren et al., 2017; Muthusamy et al., 2018; Shah et al., 2018).

## Induced Neurogenesis Through Fate Reprogramming *in vivo*

Because of the restricted neurogenesis in the adult brain and a lack of any neurogenesis in the adult spinal cord, new strategies are devised to promote neural regeneration under neuropathological conditions. One emerging regenerative strategy is to reprogram the cell fate *in vivo* for the generation of new neurons. Such *in vivo* reprogramming is largely driven by ectopic expression of fate-determining transcriptional regulators. Functionally mature new neurons can be generated through this approach in several regions of the adult CNS (Table 1).

**Table 1:** Induced neurogenesis in the adult central nervous system.

Region/Disease model	Cell source	Reprogramming factors	Functional properties of the induced neurons	References
Cortex/Stab injury; AD	Glia	*Neurod1*	Functional synapses with surrounding neurons	Guo et al. (2014)
Cortex/Stab injury	NG2 glia	*Sox2*	Synaptic inputs from innate neurons near the injury site	Heinrich et al. (2014)
Cortex/Stab injury	Glia	*Neurog2*; *Bcl-2*; α-Tocotrienol	Complex morphology	Gascon et al. (2016)
Cortex/Controlled cortical impact injury	Glia	*Oct4*; *Klf4*; *Sox2*;*c-Myc*	Electrophysiology; network-integration; reduction of tissue cavity	Gao et al. (2016)
Cortex/Stab injury	Astrocytes	*Neurod1*	Brain repair with a reduction of tissue loss	Zhang et al. (2018)
Cortex/Stab Injury	Astrocytes	*Neurog2*; *Nurr1*	Cortical layer-specific identity; synaptic connections; long axonal projections	Mattugini et al. (2019)
Cortex/Ischemic injury	Astrocytes	*Neurod1*	Synaptic connections; long-range axonal projections; improvement of motor and cognitive functions	Chen et al. (2020)
Striatum/Injection injury	Astrocytes	*SOX2*	Electrophysiology; network integration	Niu et al. (2013)
Striatum/6-OHDA lesion	Human embryonic	*Ascl1*; *Brn2a*; *Myt1l*	Innervation into host tissues	Torper et al. (2013)
	fibroblasts/human fetal lung fibroblast
Striatum/Injection injury	Astrocytes	*SOX2*	Network-integration with inputs from presynaptic neurons	Niu et al. (2015)
Striatum/Injection injury	NG2 glia	*Ascl1*; *Lmx1a*; *Nurr1*	Innervation from pre-existing local circuitry	Torper et al. (2015)
Striatum; midbrain/6-OHDA	NG2 glia	*Ascl1*; *Lmx1a*; *Nurr1*; etc.	Electrophysiology; network integration	Pereira et al. (2017)
Striatum/PD	Astrocytes	*Neurod1*; *Ascl1*; *Lmx1a*; *miR218*	Electrophysiology; rescue of spontaneous motor behavior	Rivetti di Val Cervo et al. (2017)
Striatum/PD	Astrocytes	*Ascl1*; *Pitx3*; *Lmx1a*; *Nurr1*	Electrophysiology; rescue of Parkinsonian phenotypes	Yoo et al. (2017)
Striatum/Injection injury	Striatal neurons	*SOX2*; *NURR1*; *FOXA2*; *LMX1A*; Valproic acid	Electrophysiology; network integration	Niu et al. (2018)
Striatum/Injection injury	Microglia	*Neurod1*	Integration into brain circuits with synaptic connections.	Matsuda et al. (2019)
Striatum; Cortex/Stab injury; ischemia	Non-neuronal cells	*Neurog2*; growth factors	Region-specific differentiation	Grande et al. (2013)
Dorsal midbrain; Striatum; Cortex/Stab or injection injury	Astrocytes	*Ascl1*	Electrophysiology; network integration	Liu et al. (2015)
Dentate gyrus/AD	Astrocytes	*microRNA-302/367*	Electrophysiology; improvement of spatial learning and memory	Ghasemi-Kasman et al. (2018)
Spinal cord/SCI	Astrocytes	*SOX2*	Synapse-forming interneurons	Su et al. (2014)
Spinal cord/SCI	Astrocytes	*SOX2*	Synaptic connections with local neurons	Wang L. L. et al. (2016)
Retina/Retinal injury	Müller glia	*Ascl1*	Amacrine, bipolar, or photoreceptors	Ueki et al. (2015)
Retina/Retinal injury	Müller glia	*Ascl1*; Trichostatin-A	Inner retinal neurons; synapses with host retinal neurons; response to light	Jorstad et al. (2017)
Retina/Congenital blindness	Müller glia	β-catenin; *Otx2*; *Crx*; *Nrl*	Rod photoreceptors; restoration of visual responses	Yao et al. (2018)

### Induced Neurons After Brain Injury

Through *in vivo* screens of eight transcription factors (*ASCL1*, *BRN2*, *KLF4*, *MYC*, *MYT1L*, *OCT4*, *SOX2*, and *ZFP521*) and four microRNAs (*miR9*, *miR124*, *miR125*, and *miR128*), the stem cell factor *SOX2* was identified to be sufficient to reprogram resident astrocytes into functional neurons in the adult mouse striatum (Niu et al., 2013). These induced neurons fire action potentials and make synaptic connections with other local neurons. Subsequent studies revealed that *SOX2*-mediated *in vivo* reprogramming passes through an expandable neural progenitor stage that is capable of generating multiple neurons from a single reprogrammed glia (Niu et al., 2015). Mechanistically, *SOX2* induces the expression of transcription factors *Ascl1* and *Tlx*, each of which is essential to the reprogramming process (Islam et al., 2015; Niu et al., 2015). In addition to astrocytes, *Sox2* alone is also sufficient to convert cortical NG2 glia into neurons especially following stab wound injury (Heinrich et al., 2014).

Glial cells can also be directly converted into neurons without passing through an expandable progenitor stage in the adult brain after injury. Such reprogramming is achieved by ectopic expression of transcription factors that are shown to play key roles during normal neurogenesis. Retrovirus-mediated expression of *Neurod1* can efficiently convert cortical astrocytes or NG2 glia into functional neurons after brain injury (Guo et al., 2014). Very interestingly, cortical astrocytes were mainly converted into glutamatergic neurons, while NG2 glia were reprogrammed into both glutamatergic and GABAergic neurons. On the other hand, striatal microglia was recently shown to be reprogrammed by *Neurod1*-expressing lentivirus into DARPP32+ medium spiny neurons (Matsuda et al., 2019). Together, these results indicate that cellular context has a great influence on the identity of the induced neurons.

Recent data further showed that the efficiency of *Neurod1*-mediated *in vivo* reprogramming of astrocytes could be significantly enhanced when *Neurod1* was delivered through an AAV system (Zhang et al., 2018; Chen et al., 2020). Both endothelin1-induced ischemia cortical stroke and stab wound-induced cortical injury were shown to be nearly completely repaired. Remarkably, the regenerated neurons exhibited cortical layer-specific identities, formed functional neural circuits and rescued motor and memory deficits. *Neurod1*-mediated efficient neuronal conversion of astrocytes also leads to the regeneration of beneficial astrocytes, restoration of blood-brain-barriers, and reduction of neuroinflammation. Such broad and remarkable effects of *Neurod1*-dependent reprogramming of resident astrocytes, if confirmed, will revolutionize the therapeutic strategies for brain injuries.

Other neurogenic factors including *Neurog2* and *Ascl1* are also individually capable of inducing new neurons in the adult mouse brain (Grande et al., 2013; Liu et al., 2015; Gascon et al., 2016). The *Neurog2*-mediated *in vivo* reprogramming could be significantly enhanced by local exposure to growth factors or by co-expression of *Bcl-2* and anti-oxidative treatments (Grande et al., 2013; Gascon et al., 2016). The latter treatments were revealed to act through inhibition of the lipid peroxidation-mediated ferroptosis pathway (Gascon et al., 2016). In addition to single factor-mediated fate reprogramming, the combination of *Ascl1* with *Brn2* and *Myt1l* through AAV-mediated expression also led to the conversion of striatal astrocytes or NG2 glia to neurons that could functionally integrate into the local circuits (Torper et al., 2013; Pereira et al., 2017). Upon stab wound injury, AAV-mediated expression of *Neurog2* and *Nurr1* in a Cre-dependent manner can robustly convert cortical astrocytes into functional pyramidal neurons (Mattugini et al., 2019). Remarkably, these induced pyramidal neurons exhibit cortical layer-specific identity indicated by lamina-specific marker expression and long-distance axonal projections. It will be extremely interesting to investigate whether these new cortical neurons can lead to functional improvements after TBI.

After TBI and retrovirus-mediated ectopic expression of the reprogramming OSKM factors (*Oct4*, *Sox2*, *Klf4*, and *cMyc*), cells resembling induced pluripotent stem cells (iPSCs) could be induced from reactive glial cells in the injured cortex (Gao et al., 2016). These cells extensively proliferated and spontaneously differentiated into neural stem cells and neurons. Such extensive proliferation and neural differentiation of iPSCs may be needed to fill in the cavity caused by TBI; however, tumorigenesis and simultaneous generation of non-neural cells in the brain need to be prevented for this approach to be useful for brain repair.

### Induced Neurons for Alzheimer’s Disease

Because of the progressive and pleiotropic neurodegeneration observed in AD, local induction of new neurons as a therapeutic approach may be challenging for this type of neurologic disease. Nonetheless, *in vivo* reprogramming of reactive glial cells into neurons were attempted in mouse models of AD. Similar to acute brain injuries, AD and other neurodegenerative diseases are frequently accompanied by reactive astrocytosis. The reactive astrocytes were capable of being reprogrammed into neurons by ectopic expression of *Neurod1* in the cortex of 5xFAD mice (Guo et al., 2014). Electrophysiological recordings of induced neurons in 5xFAD brain slices showed that the induced neurons were functional and made synaptic connections with surrounding neurons. Interestingly, the number of *Neurod1*-induced neurons was higher in aged and diseased brains than in young and healthy ones, suggesting that more reactive glial cells observed in aged and diseased brains might provide more cells for reprogramming *in vivo* (Guo et al., 2014). In another AD mouse model that was induced by intraventricular injection of streptozocin, *miR-302/367* ectopic expression in combination with valproate treatment was reported to induce neurons from reactive astrocytes in the dentate gyrus (Ghasemi-Kasman et al., 2018). Such treatments also led to improved performance on a behavioral task measuring spatial learning and memory, implicating a new therapeutic strategy for AD patients.

### Induced Neurons for Parkinson’s Disease

The cellular basis for PD is the loss of dopaminergic neurons in the midbrain. Transplantation of this type of neurons ameliorated many pathological symptoms associated with PD. This raised the possibility that new dopaminergic neurons derived from fate reprogramming *in vivo* may be a therapeutic alternative to cell transplantation. Several studies explored the feasibility of inducing new dopaminergic neurons in the adult mouse brains. A combination of *Ascl1*, *Lmx1a*, and *Nurr1* (ALN) was shown to be sufficient to reprogram mouse fibroblasts and astrocytes into dopaminergic neurons in culture (Addis et al., 2011; Caiazzo et al., 2011). When this combination was introduced into the adult mouse striatum, however, it completely failed to induce any dopaminergic neurons from either astrocytes or NG2 glia (Torper et al., 2015). Subsequent studies further revealed that ALN or many other combinations of fate-determining factors also failed to induce dopaminergic neurons, although they could very robustly convert resident NG2 glia into interneurons in the striatum (Pereira et al., 2017).

In contrast, in a mouse PD model that was induced by 6-hydroxydopamine (6-OHDA), striatal astrocytes were reported to be directly converted into dopaminergic neurons when a cocktail of four transcription factors (*Neurod1*, *Ascl1*, *Lmx1a*, and *miR218*) was delivered into the ipsilateral striatum (Rivetti di Val Cervo et al., 2017). The *in vivo* induced dopaminergic neurons were excitable and ameliorated some aspects of motor deficits such as gait impairments. Using a different set of transcription factors (*Ascl1*, *Pitx3*, *Lmx1a*, and *Nurr1*) and in combination with electromagnetic gold nanoparticles (AuNPs) and the presence of specific electromagnetic fields, Yoo et al. (2017) reported that dopaminergic neurons could be converted from resident striatal astrocytes in MPTP- or 6-OHDA-induced PD mouse models. Importantly, such combinatorial treatments also alleviated locomotor deficits that were observed in these mouse PD models. Results of the above two reports showed the therapeutic potential of *in vivo* reprogrammed dopaminergic neurons for PD.

Taking a slightly different strategy, Niu et al. (2018) reported that functional dopaminergic neurons could also be generated in the adult mouse striatum. Unexpectedly, systematic lineage mappings in several transgenic mouse lines rather discovered that these induced dopaminergic neurons were originated from resident striatal neurons but not from any glial cells. Such phenotypic reprogramming revealed unprecedented plasticity of mature neurons and implicated an alternative strategy to rewire brain circuits in the adult. It will be interesting to determine the biological effect of such reprogrammed dopaminergic neurons in PD.

### Induced Neurons After Spinal Cord Injury

Spinal cord injuries (SCI) lead to the disruption of neural circuits and the loss of propriospinal neurons. The addition of new neurons may well serve as relays that could form neural circuits for functional improvements. Ectopic expression of *SOX2* induced DCX+ neuroblasts in the adult spinal cord after injury (Su et al., 2014). Lineage tracing confirmed an astrocyte origin for the induced neuroblasts. These neuroblasts could expand through proliferation and eventually become mature neurons that formed connections with spinal motor neurons. Through *in vivo* screens of additional 17 factors, the p53-p21 signaling pathway was identified to be critical for controlling the expansion of *SOX2*-induced neuroblasts in the adult spinal cord (Wang L. L. et al., 2016). Downregulation of either p53 or p21 led to a greatly increased number of *SOX2*-induced neuroblasts and mature neurons. The ability to expand the number of induced neurons may be therapeutically advantageous since a SCI frequently leads to the death of many propriospinal neurons. Interestingly, a majority of these *SOX2*-induced neurons are VGLUT2+ excitatory interneurons (Wang L. L. et al., 2016), which are in sharp contrast to those in the striatum that are mainly GABAergic interneurons (Niu et al., 2015). VGLUT2+ interneurons are essential components of the locomotor circuitry and play an important role in the proper organization of the spinal locomotor network (Borgius et al., 2014). As such, it is conceivable that the induced VGLUT2 excitatory interneurons may well be capable of forming relay circuits (Courtine et al., 2008; Abematsu et al., 2010) with ascending and descending pathways that are frequently disrupted by SCI.

### Induced Neurons for Retinopathy

Müller glial cells (MGs) are the major glial cells in the retina, an integral part of the mammalian CNS that lacks any neurogenesis under both physiological and pathological conditions. Ectopic *Ascl1* in young transgenic mice renders MGs to generate retinal neurons including amacrine and bipolar cells and photoreceptors after injury (Ueki et al., 2015). However, such *Ascl1*-mediated reprogramming of MGs is restricted to the first 2 weeks after birth. An epigenetic barrier may account for the reprogramming failure of adult MGs since the chromatin is less accessible in these cells than those young MGs (Ueki et al., 2015). Indeed, when treated with a histone deacetylase inhibitor that leads to increased histone acetylation and an open chromatin structure, ectopic *Ascl1* can reprogram MGs to generate retinal neurons after retinal injury in the adult mice (Jorstad et al., 2017). Importantly, these induced neurons can make synapses with resident neurons and respond to light stimulation, indicating functional integration into the neural network. It is not clear, though, why the induce neurons are mainly bipolar cells, since lineage tracing shows that *Ascl1*-expressing progenitors can give rise to multiple neuronal subtypes during retinogenesis (Brzezinski et al., 2011).

Taking a two-step approach, Yao et al. (2018) show that activated MGs can be directly reprogrammed into functional rod photoreceptors in the adult mice. AAV-mediated ectopic expression of β-catenin enables the adult MGs to proliferate without prior injury (Yao et al., 2016). However, a majority of these activated MGs undergo cell death and rarely generate any new retinal neurons. Remarkably, subsequent AAV-mediated expression of *Otx2*, *Crx*, and *Nrl* in these activated MGs allows them to produce rod photoreceptors, which are capable of restoring visual responses in a mouse model of congenital blindness (Yao et al., 2018). It will be certainly interesting to examine whether other retinal cell types, such as retinal ganglion cells, can be similarly reprogrammed from MGs by using a different set of fate-determining factors.

## The Influence of Cellular Context on Fate Reprogramming

Emerging evidence indicates that cellular contexts, such as senescence, mitochondria dynamics, and autophagy, play critical roles during the reprogramming process.

### Senescence

Senescence is characterized by permanent cell cycle arrest. Cells in senescence secrete inflammatory cytokines, chemokines, and growth factors that are together referred to as the senescence-associated secretory phenotype (SASP; Childs et al., 2015). Senescence plays critical roles in tissue repair, embryonic development, as well as aging-related diseases (Loeser, 2009; Storer et al., 2013; Demaria et al., 2014). In a transgenic mouse model expressing the reprogramming OSKM factors for iPSCs, the extent of cell senescence is found to be positively correlated with the efficiency of cell reprogramming *in vivo* (Mosteiro et al., 2016). OSKM-induced senescent cells secrete interleukin-6, which creates a favorable microenvironment that facilitates reprogramming in adult mice. Mechanistic studies further reveal that Ink4a is critical for the induction of cell senescence and reprogramming; however, it is dispensable when p53 is removed (Mosteiro et al., 2018). Consistent with a positive role of cell senescence, the frequency of OSKM-induced reprogramming is increased in aged mice (Mosteiro et al., 2016).

### Mitochondrial Dynamics

Mitochondrion plays critical roles in many cellular processes, such as metabolism, energy production, generation of reactive oxygen species (ROS), cell signaling, and apoptosis (Sarsour et al., 2009; Osellame et al., 2012). It is also emerging as a key player in cell fate determination, maintenance of pluripotency, and cell reprogramming (Folmes et al., 2012; Maryanovich and Gross, 2013; Prieto and Torres, 2017). Mitochondrial respiratory dysfunction triggered by mutant mtDNAs blocks cellular reprogramming, though it does not affect the maintenance of the pluripotent state (Yokota et al., 2015). Direct reprogramming of somatic cells to neurons induces a metabolic switch that leads to high levels of oxidative stress and ferroptosis-dependent cell death. As such, reduction of ROS through *Bcl-2* ectopic expression or treatments with antioxidants potently promote neuronal reprogramming both in culture and in a mouse model of TBI (Gascon et al., 2016). On the other hand, mitochondrial fission accompanies the early phase of cell reprogramming (Prieto et al., 2016). Blocking the pro-fission factor *Drp1* hinders the production of iPSCs. It should be interesting to examine whether mitochondria dynamics are associated with neural reprogramming in the adult CNS.

### Autophagy

Autophagy is a cellular process that degrades cytoplasmic components and organelles by the lysosome to maintain cellular homeostasis (Mizushima and Levine, 2010). Recent *in vitro* and *in vivo* studies reveal critical roles of autophagy in the regulation of adult neurogenesis and reprogramming (Tang, 2013; Wang et al., 2015). Conditional deletion of *Fip200*, an essential gene for the induction of mammalian autophagy, leads to impaired autophagy, increased mitochondrial number, and elevated ROS in postnatal mouse neural stem cells. Consequently, stem cell maintenance and neuronal differentiation are severely impaired in these mice; and, such impairments can be rescued by reducing ROS levels through treatments with an antioxidant (Wang et al., 2013). *Beclin1*, a gene required for autophagosome formation, is similarly required for adult neurogenesis in the lateral ventricle (Yazdankhah et al., 2014). On the other hand, iPSC reprogramming requires mitochondrial clearance mediated by the AMPK-dependent but *Atg5*-independent autophagic process (Ma et al., 2015). It remains to be determined what roles of autophagy play during neural reprogramming *in vivo*.

## Issues to Consider

Despite the recent major progresses, several key issues require considerations to move forward the field of neural reprogramming *in vivo*.

### Cell Origin for Reprogrammed Neurons

The cell origin for the induced neurons requires thorough investigation and confirmation. Unlike *in vitro* reprogramming that can start from a relatively pure population of cells and the fate-switch can be directly observed under a microscope, *in vivo* reprogramming occurs in a very complex microenvironment consisting of multiple cell types including resident neurons. It is essential to use well-established lineage tracing methods or time-lapse imaging to follow the reprogramming process and confirm the cell origin for the induced neurons (Niu et al., 2013, 2018; Heinrich et al., 2014; Pilz et al., 2018). If the induced neurons originate from reactive and proliferative glial cells, the BrdU- or EdU-based labeling method is a simple and validated way to confirm that the induced neurons are indeed newly generated (Grande et al., 2013; Niu et al., 2013; Heinrich et al., 2014; Wang L. L. et al., 2016; Mattugini et al., 2019). Based on our years’ experiences working with lentiviruses and AAVs, it is not reliable to use the virus-based reporters as a sole tracing method for *in vivo* induced neurons. Despite using cell-type-specific promoters (such as the human *GFAP* promoter), the attached sequences (such as GFP vs. reprogramming factors) can significantly affect cell specificity, consistent with a previous report in transgenic mice (Su et al., 2004). Retroviruses, on the other hand, can induce fusion of virus-transduced microglia with resident mature neurons (Ackman et al., 2006). As such, experiments should be conducted to exclude the possibility that resident neurons are mistakenly considered as induced neurons.

### Molecular and Cellular Mechanisms Underlying Neural Reprogramming *in vivo*

Emerging evidence indicates that multiple pathways regulate the *in vivo* reprogramming process. The neurogenic factor *Ascl1* and the nuclear receptor *Tlx* are found to be essential mediators of *SOX2*-dependent reprogramming of astrocytes in the adult brain (Niu et al., 2015). Through a series of *in vivo* screens, the p53-p21 pathway was identified to function as a critical checkpoint for glial reprogramming in the adult mouse spinal cord (Wang L. L. et al., 2016). *Neurog2*-mediated direct neuronal reprogramming can be remarkably promoted by a cocktail of growth factors or coexpression of *Bcl-2* and anti-oxidative treatments in the adult brain (Grande et al., 2013; Gascon et al., 2016). Reprogramming of microglia by *Neurod1* is accompanied by changes in the epigenetic landscape, which gradually leads to loss of microglial traits and acquisition of neuronal identity (Matsuda et al., 2019). Inhibition of histone deacetylases, which promotes chromatin accessibility, is required for *Ascl1* to convert adult MGs into neurons in the injured retina (Jorstad et al., 2017). With recent advancements in Omics tools, such as scRNA-seq, ChIP-seq, and ATAC-seq, more molecular details on the reprogramming process are expected to emerge shortly. Cautions should be taken, however, when extrapolating results from cell culture models, since many of them might not be reproducible under *in vivo* conditions. For example, both mouse fibroblasts and astrocytes can be efficiently reprogrammed into dopaminergic neurons in culture (Addis et al., 2011; Caiazzo et al., 2011). These same reprogramming factors, nonetheless, completely failed to induce any dopaminergic neurons from glial cells in the adult mouse brain (Torper et al., 2015; Pereira et al., 2017).

Intriguingly, various glial cells can be efficiently reprogrammed by diverse factors into brain region-specific neuronal subtypes. For example, a combination of *Neurog2* and *Nurr1* reprograms adult astrocytes into diverse neuronal subtypes with cortical layer-specificity and precise long-distance axonal projections (Mattugini et al., 2019). Such feats can be similarly accomplished by ectopic expression of *Neurod1* in the adult cortex with or without prior injury (Chen et al., 2020; Liu et al., 2020). In the adult striatum, *Neurod1* can efficiently reprogram both astrocytes and microglia into mature striatal neurons (Matsuda et al., 2019; Liu et al., 2020). These striatal neurons can also be converted from oligodendrocytes by microRNA-mediated knockdown of *Ptbp1* (Weinberg et al., 2017). How do these different reprogramming factors generate similar or identical subtypes of neurons from reactive or non-reactive astrocytes, microglia, and oligodendrocytes? How is region-specificity established for the glia-converted neurons? Do these diverse glial cells retain brain region-specificity that potentially mediates such phenomenal fate-switch when a neurogenic factor is ectopically expressed? If so, how could they still maintain region- and subtype-specificity after brain injury? How could microglia, which is not even derived from the neural lineage, be efficiently reprogrammed into neuronal subtypes as those from astrocytes by the same reprogramming factor such as *Neurod1*? Since most, if not all, of the developmental cues for axon guidance, are diminished in the adult CNS, how do these newly reprogrammed neurons make precise axonal projections especially after neural injuries? Answers to these questions will provide critical insights into the cellular mechanisms for neural reprogramming *in vivo*.

### Biological Functions of Neural Reprogramming

*In vivo* neural reprogramming has shown promising biological functions for stroke-induced brain injury (Chen et al., 2020), chemical-induced PD (Rivetti di Val Cervo et al., 2017; Yoo et al., 2017), and a genetic mouse model of blindness (Yao et al., 2018). If confirmed, these advancements will bring unprecedented regeneration-based therapies for many other neural injuries and neurological diseases. Nonetheless, these new neurons may not lead to the recovery of lost memories, although they may help form new ones. It remains unclear what role of newly reprogrammed neurons play during functional recovery since the reprogramming of glial cells not only produces new neurons but also alters the microenvironment. After an injury or under degenerative conditions, reactive gliosis play critical roles in modulating tissue damage and neural regeneration (Okada et al., 2006; Sofroniew, 2009; Robel et al., 2011; Karimi-Abdolrezaee and Billakanti, 2012). Of note, scar formation and secretion of chondroitin sulfate proteoglycans (CSPG) by reactive glial cells are inhibitory for functional improvement post neural injury. Attenuation of reactive gliosis or reducing CSPG activity improves post-traumatic regeneration (Wilhelmsson et al., 2004; Lang et al., 2015), whereas increasing reactive gliosis worsens brain injuries (Mori et al., 2008). As such, it will be interesting to tease out the respective contributions of new neurons and the environmental changes to functional recovery post injuries or degenerations. On the other hand, it also remains to be determined whether glia-converted neurons play any detrimental effect, since these new neurons may well lead to disruption of preexisting neural circuits or formation of abnormal ones. Rehabilitation may be needed for the new neurons to incorporate into functional neural circuits.

## Concluding Remarks

*In vivo* neural reprogramming has achieved impressive progress, ranging from generation of diverse glia-converted neurons in multiple CNS regions to functional improvements for certain neurological conditions. Such a reprogramming-based approach may kill two birds with one stone: regeneration of functional neurons and modulation of pathological microenvironment. Nonetheless, efforts should be taken to vigorously validate the cell origin for the claimed new neurons and to tease out the molecular and cellular mechanisms underlying the reprogramming progress. The results of these efforts will lay a solid scientific foundation to move *in vivo* cell fate reprogramming towards neural repair.

## Author Contributions

WT and C-LZ wrote the manuscript and X-MX commented on the manuscript.

## Conflict of Interest

The authors declare that the research was conducted in the absence of any commercial or financial relationships that could be construed as a potential conflict of interest.

## References

[B1] Abematsu M. Tsujimura K. Yamano M. Saito M. Kohno K. Kohyama J.. (2010). Neurons derived from transplanted neural stem cells restore disrupted neuronal circuitry in a mouse model of spinal cord injury. J. Clin. Invest. 120, 3255–3266. 10.1172/jci42957 20714104 PMC2929730

[B2] Ackman J. B. Siddiqi F. Walikonis R. S. LoTurco J. J. (2006). Fusion of microglia with pyramidal neurons after retroviral infection. J. Neurosci. 26, 11413–11422. 10.1523/JNEUROSCI.3340-06.2006 17079670 PMC6674527

[B3] Addis R. C. Hsu F. C. Wright R. L. Dichter M. A. Coulter D. A. Gearhart J. D. (2011). Efficient conversion of astrocytes to functional midbrain dopaminergic neurons using a single polycistronic vector. PLoS One 6:e28719. 10.1371/journal.pone.0028719 22174877 PMC3235158

[B4] Anderson M. A. Burda J. E. Ren Y. Ao Y. O’Shea T. M. Kawaguchi R.. (2016). Astrocyte scar formation aids central nervous system axon regeneration. Nature 532, 195–200. 10.1038/nature17623 27027288 PMC5243141

[B5] Barker R. A. Götz M. Parmar M. (2018). New approaches for brain repair-from rescue to reprogramming. Nature 557, 329–334. 10.1038/s41586-018-0087-1 29769670

[B6] Barnabé-Heider F. Göritz C. Sabelström H. Takebayashi H. Pfrieger F. W. Meletis K.. (2010). Origin of new glial cells in intact and injured adult spinal cord. Cell Stem Cell 7, 470–482. 10.1016/j.stem.2010.07.014 20887953

[B7] Borgius L. Nishimaru H. Caldeira V. Kunugise Y. Low P. Reig R.. (2014). Spinal glutamatergic neurons defined by EphA4 signaling are essential components of normal locomotor circuits. J. Neurosci. 34, 3841–3853. 10.1523/JNEUROSCI.4992-13.2014 24623763 PMC6705281

[B8] Brzezinski J. A. T. Kim E. J. Johnson J. E. Reh T. A. (2011). Ascl1 expression defines a subpopulation of lineage-restricted progenitors in the mammalian retina. Development 138, 3519–3531. 10.1242/dev.064006 21771810 PMC3143566

[B9] Caiazzo M. Dell’Anno M. T. Dvoretskova E. Lazarevic D. Taverna S. Leo D.. (2011). Direct generation of functional dopaminergic neurons from mouse and human fibroblasts. Nature 476, 224–227. 10.1038/nature10284 21725324

[B12] Chen Y. C. Ma N. X. Pei Z. F. Wu Z. Do-Monte F. H. Keefe S.. (2020). A NeuroD1 AAV-based gene therapy for functional brain repair after ischemic injury through *in vivo* astrocyte-to-neuron conversion. Mol. Ther. 28, 217–234. 10.1016/j.ymthe.2019.09.003 31551137 PMC6952185

[B10] Chen G. Wernig M. Berninger B. Nakafuku M. Parmar M. Zhang C. L. (2015). *In vivo* reprogramming for brain and spinal cord repair. eNeuro 2:ENEURO.0106-15.2015. 10.1523/eneuro.0106-15.2015 26730402 PMC4699832

[B13] Childs B. G. Durik M. Baker D. J. van Deursen J. M. (2015). Cellular senescence in aging and age-related disease: from mechanisms to therapy. Nat. Med. 21, 1424–1435. 10.1038/nm.4000 26646499 PMC4748967

[B14] Courtine G. Song B. Roy R. R. Zhong H. Herrmann J. E. Ao Y.. (2008). Recovery of supraspinal control of stepping *via* indirect propriospinal relay connections after spinal cord injury. Nat. Med. 14, 69–74. 10.1038/nm1682 18157143 PMC2916740

[B15] Demaria M. Ohtani N. Youssef S. A. Rodier F. Toussaint W. Mitchell J. R.. (2014). An essential role for senescent cells in optimal wound healing through secretion of PDGF-AA. Dev. Cell 31, 722–733. 10.1016/j.devcel.2014.11.012 25499914 PMC4349629

[B16] Desplats P. Spencer B. Crews L. Pathel P. Morvinski-Friedmann D. Kosberg K.. (2012). α-Synuclein induces alterations in adult neurogenesis in Parkinson disease models *via* p53-mediated repression of Notch1. J. Biol. Chem. 287, 31691–31702. 10.1074/jbc.m112.354522 22833673 PMC3442504

[B17] Dixon K. J. (2017). Pathophysiology of traumatic brain injury. Phys. Med. Rehabil. Clin. N. Am. 28, 215–225. 10.1016/j.pmr.2016.12.001 28390509

[B18] Donovan M. H. Yazdani U. Norris R. D. Games D. German D. C. Eisch A. J. (2006). Decreased adult hippocampal neurogenesis in the PDAPP mouse model of Alzheimer’s disease. J. Comp. Neurol. 495, 70–83. 10.1002/cne.20840 16432899

[B19] Folmes C. D. Dzeja P. P. Nelson T. J. Terzic A. (2012). Metabolic plasticity in stem cell homeostasis and differentiation. Cell Stem Cell 11, 596–606. 10.1016/j.stem.2012.10.002 23122287 PMC3593051

[B20] Freire M. A. (2012). Pathophysiology of neurodegeneration following traumatic brain injury. West Indian Med. J. 61, 751–755. 23620976

[B21] Gao X. Wang X. Xiong W. Chen J. (2016). *In vivo* reprogramming reactive glia into iPSCs to produce new neurons in the cortex following traumatic brain injury. Sci. Rep. 6:22490. 10.1038/srep22490 26957147 PMC4783661

[B22] Gascon S. Murenu E. Masserdotti G. Ortega F. Russo G. L. Petrik D.. (2016). Identification and successful negotiation of a metabolic checkpoint in direct neuronal reprogramming. Cell Stem Cell 18, 396–409. 10.1016/j.stem.2015.12.003 26748418

[B23] Ghasemi-Kasman M. Shojaei A. Gol M. Moghadamnia A. A. Baharvand H. Javan M. (2018). miR-302/367-induced neurons reduce behavioral impairment in an experimental model of Alzheimer’s disease. Mol. Cell. Neurosci. 86, 50–57. 10.1016/j.mcn.2017.11.012 29174617

[B24] Gitler A. D. Dhillon P. Shorter J. (2017). Neurodegenerative disease: models, mechanisms, and a new hope. Dis. Model Mech. 10, 499–502. 10.1242/dmm.030205 28468935 PMC5451177

[B25] Grande A. Sumiyoshi K. López-Juárez A. Howard J. Sakthivel B. Aronow B.. (2013). Environmental impact on direct neuronal reprogramming *in vivo* in the adult brain. Nat. Commun. 4:2373. 10.1038/ncomms3373 23974433 PMC3786770

[B26] Guo Z. Zhang L. Wu Z. Chen Y. Wang F. Chen G. (2014). *In vivo* direct reprogramming of reactive glial cells into functional neurons after brain injury and in an Alzheimer’s disease model. Cell Stem Cell 14, 188–202. 10.1016/j.stem.2013.12.001 24360883 PMC3967760

[B27] Hayta E. Elden H. (2018). Acute spinal cord injury: a review of pathophysiology and potential of non-steroidal anti-inflammatory drugs for pharmacological intervention. J. Chem. Neuroanat. 87, 25–31. 10.1016/j.jchemneu.2017.08.001 28803968

[B28] He X. J. Nakayama H. (2015). Transiently impaired neurogenesis in MPTP mouse model of Parkinson’s disease. Neurotoxicology 50, 46–55. 10.1016/j.neuro.2015.07.007 26215120

[B29] Heinrich C. Bergami M. Gascon S. Lepier A. Viganò F. Dimou L.. (2014). Sox2-mediated conversion of NG2 glia into induced neurons in the injured adult cerebral cortex. Stem Cell Reports 3, 1000–1014. 10.1016/j.stemcr.2014.10.007 25458895 PMC4264057

[B30] Horky L. L. Galimi F. Gage F. H. Horner P. J. (2006). Fate of endogenous stem/progenitor cells following spinal cord injury. J. Comp. Neurol. 498, 525–538. 10.1002/cne.21065 16874803 PMC2553041

[B31] Horner P. J. Power A. E. Kempermann G. Kuhn H. G. Palmer T. D. Winkler J.. (2000). Proliferation and differentiation of progenitor cells throughout the intact adult rat spinal cord. J. Neurosci. 20, 2218–2228. 10.1523/JNEUROSCI.20-06-02218.2000 10704497 PMC6772504

[B32] Islam M. M. Smith D. K. Niu W. Fang S. Iqbal N. Sun G.. (2015). Enhancer analysis unveils genetic interactions between TLX and SOX2 in neural stem cells and *in vivo* reprogramming. Stem Cell Reports 5, 805–815. 10.1016/j.stemcr.2015.09.015 26607952 PMC4649261

[B33] Jessberger S. Parent J. M. (2015). Epilepsy and adult neurogenesis. Cold Spring Harb. Perspect. Biol. 7:a020677. 10.1101/cshperspect.a020677 26552418 PMC4665072

[B34] Jin K. Galvan V. Xie L. Mao X. O. Gorostiza O. F. Bredesen D. E.. (2004a). Enhanced neurogenesis in Alzheimer’s disease transgenic (PDGF-APPSw,Ind) mice. Proc. Natl. Acad. Sci. U S A 101, 13363–13367. 10.1073/pnas.0403678101 15340159 PMC516572

[B35] Jin K. Peel A. L. Mao X. O. Xie L. Cottrell B. A. Henshall D. C.. (2004b). Increased hippocampal neurogenesis in Alzheimer’s disease. Proc. Natl. Acad. Sci. U S A 101, 343–347. 10.1073/pnas.2634794100 14660786 PMC314187

[B36] Jorstad N. L. Wilken M. S. Grimes W. N. Wohl S. G. VandenBosch L. S. Yoshimatsu T.. (2017). Stimulation of functional neuronal regeneration from Muller glia in adult mice. Nature 548, 103–107. 10.1038/nature23283 28746305 PMC5991837

[B37] Karimi-Abdolrezaee S. Billakanti R. (2012). Reactive astrogliosis after spinal cord injury-beneficial and detrimental effects. Mol. Neurobiol. 46, 251–264. 10.1007/s12035-012-8287-4 22684804

[B38] Lang B. T. Cregg J. M. DePaul M. A. Tran A. P. Xu K. Dyck S. M.. (2015). Modulation of the proteoglycan receptor PTPsigma promotes recovery after spinal cord injury. Nature 518, 404–408. 10.1038/nature13974 25470046 PMC4336236

[B39] Li H. Chen G. (2016). *In vivo* reprogramming for CNS repair: regenerating neurons from endogenous glial cells. Neuron 91, 728–738. 10.1016/j.neuron.2016.08.004 27537482 PMC5466364

[B40] Liu M. H. Li W. Zheng J. J. Xu Y. G. He Q. Chen G. (2020). Differential neuronal reprogramming induced by NeuroD1 from astrocytes in grey matter versus white matter. Neural Regen. Res. 15, 342–351. 10.4103/1673-5374.265185 31552908 PMC6905344

[B41] Liu Y. Miao Q. Yuan J. Han S. Zhang P. Li S.. (2015). Ascl1 converts dorsal midbrain astrocytes into functional neurons *in vivo*. J. Neurosci. 35, 9336–9355. 10.1523/JNEUROSCI.3975-14.2015 26109658 PMC6605193

[B42] Loeser R. F. (2009). Aging and osteoarthritis: the role of chondrocyte senescence and aging changes in the cartilage matrix. Osteoarthritis Cartilage 17, 971–979. 10.1016/j.joca.2009.03.002 19303469 PMC2713363

[B43] Ma T. Li J. Xu Y. Yu C. Xu T. Wang H.. (2015). Atg5-independent autophagy regulates mitochondrial clearance and is essential for iPSC reprogramming. Nat. Cell Biol. 17, 1379–1387. 10.1038/ncb3256 26502054

[B44] Maryanovich M. Gross A. (2013). A ROS rheostat for cell fate regulation. Trends Cell Biol. 23, 129–134. 10.1016/j.tcb.2012.09.007 23117019

[B45] Matsuda T. Irie T. Katsurabayashi S. Hayashi Y. Nagai T. Hamazaki N.. (2019). Pioneer factor neurod1 rearranges transcriptional and epigenetic profiles to execute microglia-neuron conversion. Neuron 101, 472.e7–485.e7. 10.1016/j.neuron.2018.12.010 30638745

[B46] Mattugini N. Bocchi R. Scheuss V. Russo G. L. Torper O. Lao C. L.. (2019). Inducing different neuronal subtypes from astrocytes in the injured mouse cerebral cortex. Neuron 103, 1086.e5–1095.e5. 10.1016/j.neuron.2019.08.009 31488328 PMC6859713

[B47] Meletis K. Barnabé-Heider F. Carlén M. Evergren E. Tomilin N. Shupliakov O.. (2008). Spinal cord injury reveals multilineage differentiation of ependymal cells. PLoS Biol. 6:e182. 10.1371/journal.pbio.0060182 18651793 PMC2475541

[B48] Mizushima N. Levine B. (2010). Autophagy in mammalian development and differentiation. Nat. Cell Biol. 12, 823–830. 10.1038/ncb0910-823 20811354 PMC3127249

[B49] Mori T. Tan J. Arendash G. W. Koyama N. Nojima Y. Town T. (2008). Overexpression of human S100B exacerbates brain damage and periinfarct gliosis after permanent focal ischemia. Stroke 39, 2114–2121. 10.1161/strokeaha.107.503821 18451356 PMC2665284

[B50] Mosteiro L. Pantoja C. Alcazar N. Marión R. M. Chondronasiou D. Rovira M.. (2016). Tissue damage and senescence provide critical signals for cellular reprogramming *in vivo*. Science 354:aaf4445. 10.1126/science.aaf4445 27884981

[B51] Mosteiro L. Pantoja C. de Martino A. Serrano M. (2018). Senescence promotes *in vivo* reprogramming through p16^INK4a^ and IL-6. Aging Cell 17:e12711. 10.1111/acel.12711 29280266 PMC5847859

[B52] Muthusamy N. Brumm A. Zhang X. Carmichael S. T. Ghashghaei H. T. (2018). Foxj1 expressing ependymal cells do not contribute new cells to sites of injury or stroke in the mouse forebrain. Mol. Psychiatry 8:1766. 10.1038/s41598-018-19913-x 29379049 PMC5789075

[B53] Niu W. Zang T. Smith D. K. Vue T. Y. Zou Y. Bachoo R.. (2015). SOX2 reprograms resident astrocytes into neural progenitors in the adult brain. Stem Cell Reports 4, 780–794. 10.1016/j.stemcr.2015.03.006 25921813 PMC4437485

[B54] Niu W. Zang T. Wang L. L. Zou Y. Zhang C. L. (2018). Phenotypic reprogramming of striatal neurons into dopaminergic neuron-like cells in the adult mouse brain. Stem Cell Reports 11, 1156–1170. 10.1016/j.stemcr.2018.09.004 30318292 PMC6234859

[B55] Niu W. Zang T. Zou Y. Fang S. Smith D. K. Bachoo R.. (2013). *In vivo* reprogramming of astrocytes to neuroblasts in the adult brain. Nat. Cell Biol. 15, 1164–1175. 10.1038/ncb2843 24056302 PMC3867822

[B56] Obernier K. Cebrian-Silla A. Thomson M. Parraguez J. I. Anderson R. Guinto C.. (2018). Adult neurogenesis is sustained by symmetric self-renewal and differentiation. Cell Stem Cell 22, 221.e8–234.e8. 10.1016/j.stem.2018.01.003 29395056 PMC5802882

[B57] Okada S. Nakamura M. Katoh H. Miyao T. Shimazaki T. Ishii K.. (2006). Conditional ablation of Stat3 or Socs3 discloses a dual role for reactive astrocytes after spinal cord injury. Nat. Med. 12, 829–834. 10.1038/nm1425 16783372

[B58] Osellame L. D. Blacker T. S. Duchen M. R. (2012). Cellular and molecular mechanisms of mitochondrial function. Best Pract. Res. Clin. Endocrinol. Metab. 26, 711–723. 10.1016/j.beem.2012.05.003 23168274 PMC3513836

[B59] Oyinbo C. A. (2011). Secondary injury mechanisms in traumatic spinal cord injury: a nugget of this multiply cascade. Acta Neurobiol. Exp. 71, 281–299. 21731081 10.55782/ane-2011-1848

[B60] Pereira M. Birtele M. Shrigley S. Benitez J. A. Hedlund E. Parmar M.. (2017). Direct reprogramming of resident NG2 glia into neurons with properties of fast-spiking parvalbumin-containing interneurons. Stem Cell Reports 9, 742–751. 10.1016/j.stemcr.2017.07.023 28844658 PMC5599255

[B61] Pilz G. A. Bottes S. Betizeau M. Jörg D. J. Carta S. Simons B. D.. (2018). Live imaging of neurogenesis in the adult mouse hippocampus. Science 359, 658–662. 10.1126/science.aao5056 29439238 PMC6986926

[B63] Prieto J. León M. Ponsoda X. Sendra R. Bort R. Ferrer-Lorente R.. (2016). Early ERK1/2 activation promotes DRP1-dependent mitochondrial fission necessary for cell reprogramming. Nat. Commun. 7:11124. 10.1038/ncomms11124 27030341 PMC4821885

[B62] Prieto J. Torres J. (2017). Mitochondrial dynamics: in cell reprogramming as it is in cancer. Stem Cells Int. 2017:8073721. 10.1155/2017/8073721 28484497 PMC5412136

[B64] Rasband M. N. (2016). Glial contributions to neural function and disease. Mol. Cell. Proteomics 15, 355–361. 10.1074/mcp.r115.053744 26342039 PMC4739659

[B65] Ren Y. Ao Y. O’Shea T. M. Burda J. E. Bernstein A. M. Brumm A. J.. (2017). Ependymal cell contribution to scar formation after spinal cord injury is minimal, local and dependent on direct ependymal injury. Sci. Rep. 7:41122. 10.1038/srep41122 28117356 PMC5259707

[B66] Rivetti di Val Cervo P. Romanov R. A. Spigolon G. Masini D. Martín-Montañez E. Toledo E. M.. (2017). Induction of functional dopamine neurons from human astrocytes *in vitro* and mouse astrocytes in a Parkinson’s disease model. Nat. Biotechnol. 35, 444–452. 10.1038/nbt.3835 28398344

[B67] Robel S. Berninger B. Götz M. (2011). The stem cell potential of glia: lessons from reactive gliosis. Nat. Rev. Neurosci. 12, 88–104. 10.1038/nrn2978 21248788

[B68] Sarsour E. H. Kumar M. G. Chaudhuri L. Kalen A. L. Goswami P. C. (2009). Redox control of the cell cycle in health and disease. Antioxid. Redox Signal. 11, 2985–3011. 10.1089/ars.2009.2513 19505186 PMC2783918

[B69] Shah P. T. Stratton J. A. Stykel M. G. Abbasi S. Sharma S. Mayr K. A.. (2018). Single-cell transcriptomics and fate mapping of ependymal cells reveals an absence of neural stem cell function. Cell 173, 1045.e9–1057.e9. 10.1016/j.cell.2018.03.063 29727663

[B71] Smith D. K. He M. Zhang C. L. Zheng J. C. (2017). The therapeutic potential of cell identity reprogramming for the treatment of aging-related neurodegenerative disorders. Prog. Neurobiol. 157, 212–229. 10.1016/j.pneurobio.2016.01.006 26844759 PMC5848468

[B70] Smith D. K. Zhang C. L. (2015). Regeneration through reprogramming adult cell identity *in vivo*. Am. J. Pathol. 185, 2619–2628. 10.1016/j.ajpath.2015.02.025 26056931 PMC4607754

[B72] Sofroniew M. V. (2009). Molecular dissection of reactive astrogliosis and glial scar formation. Trends Neurosci. 32, 638–647. 10.1016/j.tins.2009.08.002 19782411 PMC2787735

[B73] Storer M. Mas A. Robert-Moreno A. Pecoraro M. Ortells M. C. Di Giacomo V.. (2013). Senescence is a developmental mechanism that contributes to embryonic growth and patterning. Cell 155, 1119–1130. 10.1016/j.cell.2013.10.041 24238961

[B74] Su M. Hu H. Lee Y. d’Azzo A. Messing A. Brenner M. (2004). Expression specificity of GFAP transgenes. Neurochem. Res. 29, 2075–2093. 10.1007/s11064-004-6881-1 15662842

[B75] Su Z. Niu W. Liu M. L. Zou Y. Zhang C. L. (2014). *In vivo* conversion of astrocytes to neurons in the injured adult spinal cord. Nat. Commun. 5:3338. 10.1038/ncomms4338 24569435 PMC3966078

[B76] Tang B. L. (2013). mTOR, autophagy and reprogramming. Front. Cell. Dev. Biol. 1:4. 10.3389/fcell.2013.00004 25364709 PMC4207015

[B77] Torper O. Götz M. (2017). Brain repair from intrinsic cell sources: turning reactive glia into neurons. Prog. Brain Res. 230, 69–97. 10.1016/bs.pbr.2016.12.010 28552236

[B78] Torper O. Ottosson D. R. Pereira M. Lau S. Cardoso T. Grealish S.. (2015). *In vivo* reprogramming of striatal NG2 glia into functional neurons that integrate into local host circuitry. Cell Rep. 12, 474–481. 10.1016/j.celrep.2015.06.040 26166567 PMC4521079

[B79] Torper O. Pfisterer U. Wolf D. A. Pereira M. Lau S. Jakobsson J.. (2013). Generation of induced neurons *via* direct conversion *in vivo*. Proc. Natl. Acad. Sci. U S A 110, 7038–7043. 10.1073/pnas.1303829110 23530235 PMC3637783

[B80] Ueki Y. Wilken M. S. Cox K. E. Chipman L. Jorstad N. Sternhagen K.. (2015). Transgenic expression of the proneural transcription factor Ascl1 in Muller glia stimulates retinal regeneration in young mice. Proc. Natl. Acad. Sci. U S A 112, 13717–13722. 10.1073/pnas.1510595112 26483457 PMC4640735

[B81] Urrea C. Castellanos D. A. Sagen J. Tsoulfas P. Bramlett H. M. Dietrich W. D. (2007). Widespread cellular proliferation and focal neurogenesis after traumatic brain injury in the rat. Restor. Neurol. Neurosci. 25, 65–76. 17473396

[B86] Wang X. Gao X. Michalski S. Zhao S. Chen J. (2016). Traumatic brain injury severity affects neurogenesis in adult mouse hippocampus. J. Neurotrauma 33, 721–733. 10.1089/neu.2015.4097 26414411 PMC4841001

[B82] Wang C. Liang C. C. Bian Z. C. Zhu Y. Guan J. L. (2013). FIP200 is required for maintenance and differentiation of postnatal neural stem cells. Nat. Neurosci. 16, 532–542. 10.1038/nn.3365 23542691 PMC3637881

[B84] Wang L. L. Su Z. Tai W. Zou Y. Xu X. M. Zhang C. L. (2016). The p53 pathway controls SOX2-mediated reprogramming in the adult mouse spinal cord. Cell Rep. 17, 891–903. 10.1016/j.celrep.2016.09.038 27732862 PMC5094368

[B85] Wang S. Xia P. Rehm M. Fan Z. (2015). Autophagy and cell reprogramming. Cell. Mol. Life Sci. 72, 1699–1713. 10.1007/s00018-014-1829-3 25572296 PMC11113636

[B83] Wang L. L. Zhang C. L. (2018). Engineering new neurons: *in vivo* reprogramming in mammalian brain and spinal cord. Cell Tissue Res. 371, 201–212. 10.1007/s00441-017-2729-2 29170823 PMC5750094

[B87] Weinberg M. S. Criswell H. E. Powell S. K. Bhatt A. P. McCown T. J. (2017). Viral vector reprogramming of adult resident striatal oligodendrocytes into functional neurons. Mol. Ther. 25, 928–934. 10.1016/j.ymthe.2017.01.016 28202388 PMC5383550

[B88] Wilhelmsson U. Li L. Pekna M. Berthold C. H. Blom S. Eliasson C.. (2004). Absence of glial fibrillary acidic protein and vimentin prevents hypertrophy of astrocytic processes and improves post-traumatic regeneration. J. Neurosci. 24, 5016–5021. 10.1523/JNEUROSCI.0820-04.2004 15163694 PMC6729371

[B89] Winner B. Regensburger M. Schreglmann S. Boyer L. Prots I. Rockenstein E.. (2012). Role of α-synuclein in adult neurogenesis and neuronal maturation in the dentate gyrus. J. Neurosci. 32, 16906–16916. 10.1523/JNEUROSCI.2723-12.2012 23175842 PMC4962062

[B90] Yamamoto S. Yamamoto N. Kitamura T. Nakamura K. Nakafuku M. (2001). Proliferation of parenchymal neural progenitors in response to injury in the adult rat spinal cord. Exp. Neurol. 172, 115–127. 10.1006/exnr.2001.7798 11681845

[B91] Yao K. Qiu S. Tian L. Snider W. D. Flannery J. G. Schaffer D. V.. (2016). Wnt regulates proliferation and neurogenic potential of muller glial cells *via* a Lin28/let-7 miRNA-dependent pathway in adult mammalian retinas. Cell Rep. 17, 165–178. 10.1016/j.celrep.2016.08.078 27681429 PMC5076887

[B92] Yao K. Qiu S. Wang Y. V. Park S. J. H. Mohns E. J. Mehta B.. (2018). Restoration of vision after de novo genesis of rod photoreceptors in mammalian retinas. Nature 560, 484–488. 10.1038/s41586-018-0425-3 30111842 PMC6107416

[B93] Yazdankhah M. Farioli-Vecchioli S. Tonchev A. B. Stoykova A. Cecconi F. (2014). The autophagy regulators Ambra1 and Beclin 1 are required for adult neurogenesis in the brain subventricular zone. Cell Death Dis. 5:e1403. 10.1038/cddis.2014.358 25188513 PMC4540193

[B94] Yokota M. Hatakeyama H. Okabe S. Ono Y. Goto Y. (2015). Mitochondrial respiratory dysfunction caused by a heteroplasmic mitochondrial DNA mutation blocks cellular reprogramming. Hum. Mol. Genet. 24, 4698–4709. 10.1093/hmg/ddv201 26025377

[B95] Yoo J. Lee E. Kim H. Y. Youn D. H. Jung J. Kim H.. (2017). Electromagnetized gold nanoparticles mediate direct lineage reprogramming into induced dopamine neurons *in vivo* for Parkinson’s disease therapy. Nat. Nanotechnol. 12, 1006–1014. 10.1038/nnano.2017.133 28737745

[B96] Zhang C. McNeil E. Dressler L. Siman R. (2007). Long-lasting impairment in hippocampal neurogenesis associated with amyloid deposition in a knock-in mouse model of familial Alzheimer’s disease. Exp. Neurol. 204, 77–87. 10.1016/j.expneurol.2006.09.018 17070803 PMC1853320

[B97] Zhang L. Lei Z. Guo Z. Pei Z. Chen Y. Zhang F. (2018). Reversing glial scar back to neural tissue through NeuroD1-mediated astrocyte-to-neuron conversion. bioRxiv [Preprint]. 10.1101/261438

[B98] Zhao C. Deng W. Gage F. H. (2008). Mechanisms and functional implications of adult neurogenesis. Cell 132, 645–660. 10.1016/j.cell.2008.01.033 18295581

[B99] Zheng W. ZhuGe Q. Zhong M. Chen G. Shao B. Wang H.. (2013). Neurogenesis in adult human brain after traumatic brain injury. J. Neurotrauma 30, 1872–1880. 10.1089/neu.2010.1579 21275797 PMC3815038

[B100] Ziemka-Nałecz M. Zalewska T. (2012). Endogenous neurogenesis induced by ischemic brain injury or neurodegenerative diseases in adults. Acta Neurobiol. Exp. 72, 309–324. 23377263 10.55782/ane-2012-1904

